# Predictors for the Level of Quality of Life Among Older Adults: Approaches to the Effect of Sociodemographic and Chronic Diseases

**DOI:** 10.1155/jare/5436660

**Published:** 2024-11-22

**Authors:** Khulud Ahmad Rezq, Maadiah M. Algamdi, Futun Alatawi, Danah Altamimi, Nouf Albalawi, Atheer Albalawi, Taghreed Abed, Dana Alatawi, Reem Alhthli

**Affiliations:** Community & Psychiatric Health Nursing Department, Faculty of Nursing, University of Tabuk, Tabuk, Saudi Arabia

**Keywords:** chronic illness, older adults, prevalence, quality of life, Saudi Arabia

## Abstract

**Objectives:** This cross-sectional study aimed to assess the quality of life (QOL) and chronic disease prevalence among 265 individuals over the age of 60.

**Methods:** Utilizing the quality-of-life index (QLI), data were collected through an online questionnaire from eligible participants, not mentally disabled.

**Results:** Significant variations in chronic conditions were found based on marital status, education, employment, and daily activities. QOL declined with age, divorce, unemployment, and dependency on caregivers but improved with a university degree. Living with family positively impacted overall QOL, while older age, unemployment, dependency, hypertension, and kidney disease were associated with lower QOL scores across various domains.

**Conclusion:** Sociodemographic factors and chronic conditions had pivotal role in shaping the QOL for individuals over the age of 60, emphasizing the importance of targeted interventions for improved well-being in this population.

## 1. Introduction

Aging, an inevitable natural process involving a decline in physical and mental abilities, presents varying impacts among individuals and societies World Health Organization [1]. Retirement age, typically set at 65 for males and 60 for females, signifies the early stages of old age, yet aging does not uniformly imply frailty or dependency at age 60, as the individual experiences differ. Recent statistics from the Department of Statistics in Saudi Arabia [2] indicate that there are around one million and 3000 older adults, constituting 5% of Saudi Arabia's population, currently reside in the country. Globally, projections from [3] suggest that the number of individuals over the age of 60 will double to 2.1 billion by 2050, with the number of those over 80 years old tripling to 426 million between 2020 and 2050. These trends underscore the significance of comprehending and addressing the diverse experiences and challenges associated with aging on both individual and societal levels.

The aging process has led to a rise in the prevalence of older adults experiencing multiple chronic diseases [4]. Chronic diseases, defined as conditions lasting a year or longer that require ongoing medical care and often limit daily activities, pose significant challenges to the older adults [5]. Globally, noncommunicable diseases (NCDs) contribute to 17 million premature deaths annually, with 86% occurring in low- and middle-income countries [6]. Cardiovascular diseases account for the majority of NCD-related deaths (17.9 million per year), followed by cancer (9.3 million), chronic respiratory diseases (4.1 million), and diabetes (2.0 million, including diabetes-related kidney disease deaths) [6]. In Saudi Arabia, the General Authority for Statistics (GASTAT) reported that 11.1% of the population has cardiovascular disease, 49.2% has diabetes, 1% has cancer, and 51.7% has hypertension [7]. These statistics underscore the substantial impact of chronic diseases on the health landscape, necessitating comprehensive strategies for prevention and management.

Quality of life (QOL), a complex concept encompassing physical and mental health, social and economic factors, personal perspectives, and environmental interactions, is adversely affected by chronic diseases, particularly in the older adults [8]. In Saudi Arabia, where the older adults population constitutes 5%, hypertension is the most prevalent chronic disease at 51.7%, followed by diabetes at 49% [7]. Recent findings by Alqahtani et al. [9] indicated high rates of hypertension (64%), diabetes (63.2%), and musculoskeletal conditions (49.6%) among 405 participants, underscoring the significant impact of chronic diseases on the well-being of the older adults in the country.

QOL, defined by the World Health Organization, encompasses the overall well-being of individuals and societies, considering both positive and negative aspects of life [5]. The global older adult population is increasing, with two-thirds residing in developing nations, and this trend is expected to continue, reaching nearly three-quarters by the [6]. In gerontology, the impact of chronic diseases on the QOL of older individuals is increasingly recognized. Bao et al. [10] conducted a cross-sectional study in Southern China, revealing a decline in health-related quality of life (HRQoL) among older adults, influenced by various multimorbidity patterns. The findings revealed a decline in HRQoL among the elderly, which was influenced by different types and combinations of chronic diseases, even after adjusting for confounding factors. Similarly, In Saudi Arabia, Kamal et al. [8] found that multiple diseases, high pain intensity, and chronic pain negatively affected the QOL of older adults. These studies underscore the global significance of understanding and addressing the impact of chronic diseases on the well-being of aging populations. Understanding the impact of chronic diseases on QOL is crucial as it raises awareness within society and helps in finding appropriate solutions to improve the factors affecting QOL [4]. Therefore, this study aims to evaluate the QOL and the prevalence of chronic diseases among older adults.

## 2. Method

### 2.1. Study Design and Sample

**Figure 1:**
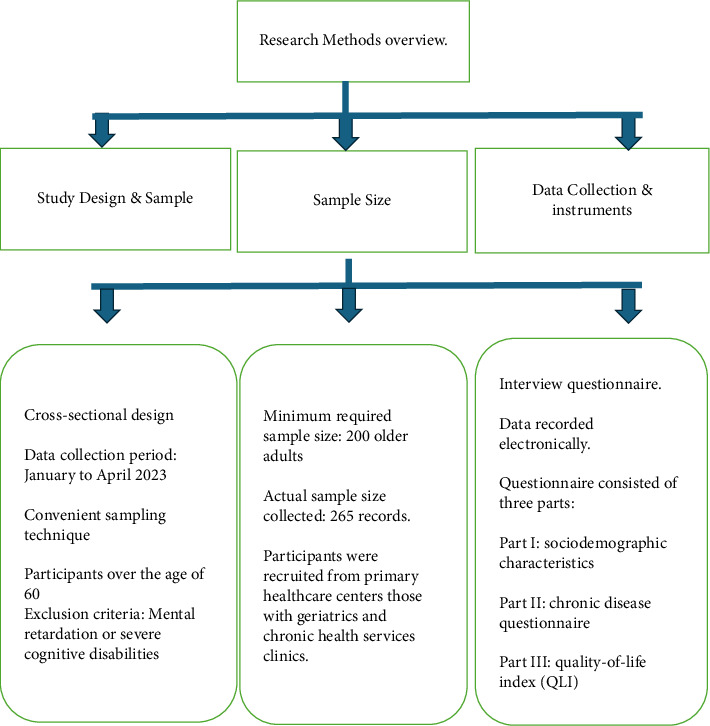
Research method overview diagram.

The study employed a cross-sectional design to evaluate the QOL and the prevalence of chronic diseases among the older adults. Data was collected between January and April 2023 using a convenient sampling technique. Participants aged 60 and above were recruited opportunistically from primary healthcare centers offering geriatrics and chronic health services. The research team identified eligible participants who attended these clinics and met the inclusion criteria, excluding those with mental retardation or severe cognitive disabilities. The research team approached potential participants to seek their consent for the study (Figure 1).

#### 2.1.1. Sample Sizes

We applied the rule of thumb for sample size calculation (https://doi.org/10.1093/aje/kwk052) since we planned to construct multiple linear regression analyses for the predictors of the QOL score. The rule indicates that at least 10 subjects are required per candidate predictor (variable). In the current study, we intended to include 8 sociodemographic variables and a total of 12 chronic conditions to account for the independent associations with the QOL (20 variables). As such, the minimum required sample size was 200 older adults. However, we sought to increase the sample size to account for the potential addition of independent predictors. Therefore, we collected a total of 265 records.

#### 2.1.2. Data Collection and Instruments

Data were collected using an interview questionnaire, and the responses were electronically recorded by the researchers. Before commencing interviews, primary researchers discussed the study's purpose with all researchers to ensure participants understood the significance of their contributions. The proper process of obtaining informed consent, including explanations of study objectives and potential benefits, was emphasized. Detailed guidance was provided on the survey administration process, focusing on accurate recording of responses, and addressing participant queries with patience and empathy. After practicing interview role-play, the interview duration was determined to be 10 min. The research questionnaires consisted of three parts: Part I encompassed sociodemographic characteristics, including age, sex, socioeconomic status, occupation, living conditions, educational level, marital status, and level of dependency. In Part II, there was a chronic disease questionnaire where we asked participants if they “have a certain chronic disease?” Yes/no, if yes, an additional question asked about the onset. Chronic diseases included in the survey were hypertension, diabetes, heart disease, kidney disease, respiratory diseases, osteoporosis, high cholesterol, eye and ear disorders, memory problems, cancer, and stroke. Part III consisted of the generic version of the quality-of-life index (QLI), developed by Ferrans and Powers [11]. The QLI measured individuals' QOL and comprised four major subscales (health and functioning subscale, psychological/spiritual subscale, social and economic subscale, and family subscale) The Arabic version of the 66-item QLI questionnaire used a 6-point Likert-type scale (i.e., very dissatisfied to very satisfied and very unimportant to very important). Scores ranged from 0 to 30, with higher scores indicating better QOL. The instrument underwent testing among the Arab population, demonstrating evidence of internal consistency [12]. Reliability estimates for the instrument were 0.94 and 0.97 [12].

### 2.2. Statistical Analysis

Statistical analysis was conducted using RStudio (R Version 4.1.1). Categorical data were summarized as frequencies and percentages, while continuous variables were presented as mean (standard deviation [SD]). To assess statistical differences in QOL across demographic groups, the Wilcoxon rank sum test was employed for variables with two categories, and the Kruskal–Wallis rank sum test was used for variables with three or more categories. Multivariate regression analyses were performed to identify independent predictors of QOL, utilizing QOL scores as dependent variables. Five separate models were constructed: one for overall QOL and four for QOL subscales. Sociodemographic characteristics and chronic conditions served as independent variables and were incorporated using a forward stepwise approach. A significance level of *p* < 0.05 was considered statistically significant.

### 2.3. Ethical Consideration

The research received ethical clearance from the University of Tabuk Ethical Committee under approval number UT-256-99-2023. Before commencing the study, all participants were presented with a consent form that clearly explained the voluntary nature of their involvement and their right to withdraw at any point. We maintained strict confidentiality of their personal information and guaranteed their privacy. The study did not pose any expected psychological, social, or physical risks to the participants, and no financial incentives were offered for their participation.

## 3. Results

The internal consistency analysis for this study indicated excellent reliability for the overall QOL survey (Cronbach's alpha = 0.978). This reliability was also observed across all subscales, including health and functioning, social and economic QOL, psychological and spiritual, and family QOL (Cronbach's alpha = 0.963, 0.921, 0.957, and 0.920, respectively). The mean (SD) QOL score for the overall sample was 20.55 (5.97).

### 3.1. Sociodemographic Characteristics

**Figure 2:**
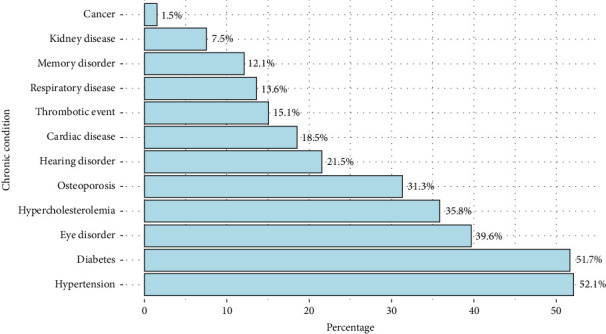
The percentages of chronic conditions among the older adults population.

Data from 265 older adults were analyzed in the current study. More than half of the sample were females (61.1%), married (61.1%), over age 60–69 years (60.0%), and had a monthly income of 3000 to 10,000 SAR (71.3%). Almost one-quarter of the population under study were illiterates (25.7%), and approximately half of them were unemployed (44.9%) or retired (48.3%). The majority of the participants were living with a family (87.2%), and 43.8% of them performed their daily activities independently (Table 1). A great proportion of the participants had at least one chronic condition (87.2%), among whom 43.3% had four conditions or more (Table 1). The most common chronic conditions among the older adults population were hypertension (52.1%), diabetes (51.7%), and eye disorders (39.6%) (Figure 2).

### 3.2. Characteristics of the QOL Scale and Subscales

The analysis of internal consistency showed that the overall QOL survey showed excellent reliability (Cronbach's alpha = 0.978). This was also applicable to all subscales, including health and functioning, social and economic, psychological, and spiritual, and family (Cronbach's alpha = 0.963, 0.921, 0.957, and 0.920, respectively). The mean (SD) QOL score of the overall sample was 20.55 (5.97). More details about the descriptive statistics of the QOL scale and subscales are provided in Table 2.

### 3.3. Differences Between Subjects With and Without Chronic Conditions

The prevalence of chronic conditions differed significantly based on marital status (*p* < 0.001), educational level (*p* < 0.001), employment status (*p*=0.031), and reliance on a person to do daily activities (*p* < 0.001, Table 3). Regarding the overall QOL score, participants with the following chronic conditions had consistently lower QOL than their peers with chronic conditions: diabetes (*p*=0.013), hypertension (*p* < 0.001), osteoporosis (*p* < 0.001), eye disorders (*p*=0.005), hypercholesterolemia (*p*=0.003), kidney disease (*p* < 0.001), hearing disorders (*p*=0.006), respiratory diseases (*p*=0.012), cardiac diseases (*p*=0.001), and having ever a thrombotic event (*p*=0.009, Table 4).

### 3.4. Factors Associated With the Scores of Overall QOL and QOL Subscales

The mean QOL scores decreased significantly with advancing age (*p* < 0.001) across all QOL subscales: health and functioning (*p* < 0.001), social and economic (*p*=0.007), and psychological/spiritual (*p* < 0.001). Divorced individuals had lower overall QOL scores than married individuals (*p*=0.004), consistent across health and functioning (*p* < 0.001), psychological/spiritual (*p*=0.030), and family subscales (*p*=0.001).

Participants with a university degree had higher QOL scores than those with other educational levels (*p*=0.004). Unemployment was associated with lower QOL scores (*p* values ranged from 0.002 to 0.027) across overall QOL, health and functioning, and social and economic scores. Living with a caregiver was linked to lower overall QOL (*p* < 0.001).

Older adults entirely dependent on others had lower QOL scores across all domains compared to partially dependent or independent individuals (*p* < 0.001). Those with at least one chronic condition had lower QOL scores (*p* values ranged from 0.008 to 0.011), with the number of chronic conditions contributing to significant differences (*p* < 0.001). Gender and monthly income did not significantly impact overall QOL or QOL subscales (*p* > 0.05) (Table 5).

### 3.5. Independent Predictors of Older Adults QOL

On the multivariate analysis, living with a family was independently associated with higher scores of overall QOL (*p*=0.041), psychological/spiritual QOL (*p*=0.021), and family QOL (*p*=0.011). Older adults over age 70–79 years independently had lower scores of health and functioning (*p*=0.023) and psychological/spiritual QOL (*p*=0.012). Overall, older individuals had significantly lower health and functioning scores (*p*=0.016). Unemployment was a significant predictor of lower scores of overall QOL (*p*=0.049), health and functioning QOL (*p*=0.043), and social and economic QOL (*p*=0.013). Older adults partially dependent on others for daily activities were more likely to have low scores of overall QOL (*p*=0.036) and health and functioning (*p*=0.007). Those entirely dependent on others had lower scores of health and functioning (*p*=0.024). Hypertension was independently associated with lower scores of overall QOL (*p*=0.010) and health and functioning (*p*=0.001). Kidney disease was a significant independent predictor of lower scores of overall QOL (*p*=0.004), health and functioning (*p*=0.009), social and economic (*p*=0.003), psychological/spiritual (*p*=0.013), and family QOL (*p*=0.023) (Table 6).

## 4. Discussion

The objective of the current study was to assess the QOL and the prevalence of chronic diseases among the older adult population. The findings of this study provide valuable demographic insights into the study population. The sample comprised predominantly females (61.1%), reflecting their higher life expectancy and healthcare-seeking behavior in this age group [13]. Zeng et al. [14] also noted that older women tend to utilize outpatient and inpatient services more frequently. Most participants were married and aged between 60 and 69 years. Approximately half of them were either unemployed or retired, and the majority lived with their families. A significant proportion of the sample reported managing daily activities independently.

This study did not find a significant difference in the prevalence of chronic conditions between males and females, indicating that gender may not be a decisive factor in the occurrence of these conditions within this sample. However, it is important to note that the higher proportion of female participants in the sample could affect the statistical power to detect gender differences. Conversely, Temkin et al. [15] observed distinct presentations of chronic conditions and a higher prevalence rate among women compared to men.

Marital status in this study showed a significant correlation with chronic conditions. Single individuals exhibited a lower prevalence (48.0%) compared to married (87.7%), divorced (93.8%), and widowed individuals (100.0%). This disparity may be attributed to varying levels of social support and stress within these groups. Perkins et al. [16] noted that widowhood negatively impacted health outcomes in women more than in men, although men widowed within the last 0–4 years had a higher risk of diabetes compared to married men. Recently, widowed women and those widowed long term were also more likely to experience psychological distress, poorer self-rated health, and hypertension.

Additionally, educational level significantly influenced chronic conditions in this study. Participants with higher education had a lower prevalence of chronic conditions (62.9%) compared to those with lower education levels, such as illiterate (98.5%) and primary school (100%). This finding is consistent with Fonseca et al. [17], who reported that more years of education lead to better health outcomes, including reduced risks of poor health, daily living difficulties, and chronic illness. These results suggest that higher education is associated with improved health literacy and healthier lifestyles, contributing to a lower prevalence of chronic conditions.

Moreover, the current study revealed a significant association between employment status and the prevalence of chronic conditions. Employed individuals had a lower prevalence of chronic conditions (66.7%) compared to retired (87.5%) and unemployed participants (89.9%). This finding aligns with Yildiz et al. [18], who found that unemployed individuals had a higher prevalence of psychological disorders, cardiovascular disease, inflammatory diseases, and respiratory illnesses compared to those who were employed. Conversely, living conditions did not significantly impact the prevalence of chronic conditions. Whether participants lived with a caregiver, family, or alone, the prevalence of chronic conditions remained relatively consistent, suggesting that living arrangements do not play a significant role in the development of chronic conditions in this sample.

Monthly income did not show a significant association with chronic conditions. However, a trend is noticeable where higher income brackets exhibit a slightly lower prevalence of chronic conditions (76.2%) compared to the lowest income bracket (87.8%). This finding aligns with Benavidez et al. [19], who reported that areas with the highest prevalence of chronic disease often face significant socioeconomic disadvantages and greater barriers to healthcare access. The observed trend in this study suggests that higher income may provide better access to healthcare and healthier living conditions, although these differences did not reach statistical significance in our sample.

Regarding health status, the findings revealed that more than half of the participants had hypertension and diabetes, while more than one-third had eye disorders. These results are consistent with a study conducted by Alqahtani et al. in Aseer, Saudi Arabia, which reported similar prevalence rates of hypertension, diabetes, and musculoskeletal diseases among participants [9]. Furthermore, the study found a significant decrease in overall QOL with advancing age (60–69, 70 to 79, and 80 years and above). This decrease was observed across various domains, including health and functioning, social and economic factors, and psychological/spiritual well-being. Additionally, divorced respondents in our study demonstrated significantly lower overall QOL scores compared to those who were married, single, or widowed. This finding aligns with previous research indicating that divorced or separated individuals, particularly women, experience notable declines in QOL, including reductions in existential well-being and social support–related QOL [20]. These differences likely stem from the sociopsychological challenges such as emotional distress, economic instability, and social stigma that divorced individuals often face.

Educational attainment also influenced QOL, as participants with a university degree reported higher QOL scores compared to those with other types of degrees. Similarly, employment status impacted QOL, with unemployed older individuals reporting significantly lower scores compared to those who were employed or retired. These findings align with a study by Ajadi et al. in Nigeria, which found that respondents with chronic illness had lower HRQoL in various domains compared to those without a chronic illness [21]. Living with a caregiver and dependency on others for daily activities were associated with lower QOL scores across all domains in our study. The presence of chronic conditions also correlated with lower QOL scores. Additionally, the number of chronic conditions showed significant differences in mean QOL scores across various domains. However, our study did not find significant differences in overall QOL or its subscales based on gender or monthly income, which is consistent with findings by Faronbi, Ajadi, and Gobbens [21], who observed a strong correlation between HRQoL and the presence of chronic illness, type of diagnosis/condition, and multimorbidity. Chantakeeree et al. conducted a study in Thailand and reported that comorbidity and perceived health status were predictors of QOL, with older adults living in cities and with families showing greater QOL compared to those living in rural areas [22]. Furthermore, our study supported Joshi's research in Nepal, indicating that older adults living with family had better overall QOL compared to those living alone [23].

Unemployment in our study was associated with lower scores in overall QOL, health and functioning, and social and economic aspects. The inability to perform daily tasks independently and increased dependence on others also contributed to lower health and functioning scores. Our study's multivariate analysis identified hypertension as independently associated with lower scores in overall QOL and health and functioning, consistent with findings by Wong, Xu, and Cheung in Hong Kong [24]. A significant portion of the older adults population in this study (43.8%) reported being independent in their daily activities, indicating that many older adults can manage their lives without assistance. This finding is consistent with Wang et al. [25], who reported an independence rate of 46.3% in men and 41.1% in women among the older adults in urban areas. Similarly, Galof and Gričar [26] found that 43.8% of the older adults reported being independent in their daily activities. Factors such as good health, social support, and access to resources enable the older adults to lead active lives. Conversely, 38.1% of the older adults population were partially dependent on their daily activities. This partial dependence may result from physical limitations, chronic health conditions, or age-related changes affecting mobility and dexterity [27]. This group experiences a significant negative impact on health and functioning, indicating that reliance on others for daily activities affects QOL. It is essential to provide support services such as home healthcare or assistive devices to help partially dependent older adults maintain a good QOL [27].

A smaller yet notable portion of the older adults population (18.1%) was entirely dependent on others for daily activities. Full dependence may be attributed to advanced age, severe health conditions, or disabilities that impede the ability to perform basic tasks. This group faces significant negative effects on health and functioning, underscoring the importance of independence for a higher QOL. Edjolo et al. [28] found that more than 90% of older adults experience a decline in functional ability, with 27% facing significant dependency in daily activities for 2–4 years before death. Specialized care and support, such as full-time caregivers, nursing homes, or long-term care facilities, are needed for entirely dependent older adults. Furthermore, this study revealed a significant association between dependency in daily activities and chronic conditions. Entirely dependent individuals had the highest prevalence of chronic conditions (97.9%), followed by partially dependent individuals (92.1%). Independence in daily activities correlated with higher QOL scores across all domains. Those who are entirely dependent on others for daily activities had significantly lower QOL scores, highlighting the impact of independence on QOL. These findings are consistent with a longitudinal study from 2013, which found that older adults with multiple chronic conditions experience more limitations in daily activities and poorer QOL [29]. Additionally, Molina-Mula et al. [30] reported that dependence is influenced by underlying health issues, mobility challenges, and daily activities, resulting in a diminished perception of QOL among patients with complex chronic conditions.

The findings of this study align with the socioecological perspectives discussed by Kim [31], particularly regarding the influence of sociodemographic factors such as education, marital status, and employment on health outcomes and QOL among older adults. Kim emphasizes the role of social structures in shaping longevity and well-being, suggesting that higher education and employment status offer protective benefits. This is consistent with the current study's results, which show that participants with higher education and those employed had a lower prevalence of chronic diseases and higher QOL. The link between education and employment with better health outcomes supports Kim's assertion that social capital and education enhance health literacy and improve access to healthcare services, fostering healthier aging.

Additionally, Kim [31] highlights the importance of social support systems, particularly family structures, in promoting longevity and QOL. The current study reflects this, as older adults who lived with their families or had stronger social support systems reported better health outcomes compared to those living alone or with less social integration. These findings underscore the need for policies that promote social inclusion and support for older adults, which can improve their QOL and survival probabilities. Overall, the results of this study demonstrate a range of dependency levels among the older adult population, from complete independence to full dependence. This variation highlights the importance of providing tailored support and resources to meet the diverse needs of older adults. Policies and programs should focus on delivering appropriate services and care options to help older adults maintain their QOL and dignity, regardless of their level of dependence. Furthermore, the study emphasizes the importance of considering multiple sociodemographic factors and implementing interventions that promote independence, family support, and effective management of chronic conditions to enhance the QOL of older adults.

### 4.1. Limitation and Strength

A notable strength of this study is the use of a survey research methodology, which is widely recognized as a reliable method of inquiry. Surveys employ standardized questions presented in a consistent manner to all participants, enhancing the potential to generate reliable and consistent results. However, this study has limitations. Firstly, its cross-sectional design restricts the ability to establish causal relationships between changes in QOL and chronic diseases among older adults. Secondly, the higher proportion of female participants (61.1%) in the sample may impact the generalizability of the findings. Future studies could benefit from randomized sampling methods to improve sample representativeness and alleviate potential gender biases.

## 5. Conclusion

This study sheds light on the relationship between QOL and chronic diseases among the older adults population in Saudi Arabia. The findings indicate a high prevalence of chronic conditions, with hypertension and diabetes being the most common. Advanced age, marital status, educational level, employment status, and level of dependency were identified as significant factors influencing QOL. Importantly, the presence of chronic diseases had a negative impact on overall QOL.

## 6. Recommendation

The study emphasizes the need for early intervention and comprehensive geriatric care to address the effects of chronic diseases on the QOL of older adults. Promoting supportive family environments, improving employment opportunities, and effective management of chronic conditions are essential for enhancing the well-being of the older adults population. Future research should focus on longitudinal studies to establish causal relationships and further explore the dynamics between chronic diseases and QOL in older adults. By understanding these factors and implementing targeted interventions, we can strive toward improving the QOL and overall well-being of the older adults in communities.

## Figures and Tables

**Table 1:** Sociodemographic characteristics.

Parameter	Category	*N* (%)
Gender	Male	103 (38.9%)
	Female	162 (61.1%)

Age	60 to 69	159 (60.0%)
	70 to 79	71 (26.8%)
	80 or more	35 (13.2%)

Marital status	Married	162 (61.1%)
	Single	25 (9.4%)
	Divorced	16 (6.0%)
	Widow	62 (23.4%)

Educational level	Illiterate	68 (25.7%)
	Primary school	50 (18.9%)
	Middle school	32 (12.1%)
	Secondary school	53 (20.0%)
	University	62 (23.4%)

Employment status	Employed	18 (6.8%)
	Retired	128 (48.3%)
	Unemployed	119 (44.9%)

Living condition	With caregiver	13 (4.9%)
	With family	231 (87.2%)
	Alone	21 (7.9%)

Rely on someone to do daily activities	Independent	116 (43.8%)
	Partially dependent	101 (38.1%)
	Entirely dependent	48 (18.1%)

Monthly income (SAR)	3000 to 10,000	189 (71.3%)
	11,000 to 20,000	55 (20.8%)
	> 20,000	21 (7.9%)

Chronic conditions	Yes	231 (87.2%)
	No	34 (12.8%)

Number of chronic conditions	1 to 3	131 (56.7%)
	4 or more	100 (43.3%)

**Table 2:** Descriptive statistics of the QOL scale and subscales.

Scale or subscale	Cronbach's alpha	Mean (SD)	Min	Max
Overall QOL	0.978	20.55 (5.97)	7.15	30.00
Health and functioning	0.963	18.94 (6.78)	3.27	30.00
Social and economic	0.921	20.12 (6.12)	6.25	30.00
Psychological and spiritual	0.957	22.09 (6.76)	6.21	30.00
Family	0.920	23.25 (6.17)	1.50	30.00

Abbreviation: SD, standard deviation.

**Table 3:** Demographic-based differences between those with and without chronic conditions.

Parameter	Category	Chronic conditions∗		*p* value
		No, *N* = 34	Yes, *N* = 231	
Gender	Male	13 (12.6%)	90 (87.4%)	0.935
	Female	21 (13.0%)	141 (87.0%)	

Age	60 to 69	25 (15.7%)	134 (84.3%)	0.100
	70 to 79	8 (11.3%)	63 (88.7%)	
	80 or more	1 (2.9%)	34 (97.1%)	

Marital status	Married	20 (12.3%)	142 (87.7%)	< 0.001
	Single	13 (52.0%)	12 (48.0%)	
	Divorced	1 (6.2%)	15 (93.8%)	
	Widow	0 (0.0%)	62 (100.0%)	

Educational level	Illiterate	1 (1.5%)	67 (98.5%)	< 0.001
	Primary school	0 (0.0%)	50 (100.0%)	
	Middle school	1 (3.1%)	31 (96.9%)	
	Secondary school	9 (17.0%)	44 (83.0%)	
	University	23 (37.1%)	39 (62.9%)	

Employment status	Employed	6 (33.3%)	12 (66.7%)	0.031
	Retired	16 (12.5%)	112 (87.5%)	
	Unemployed	12 (10.1%)	107 (89.9%)	

Living condition	With caregiver	1 (7.7%)	12 (92.3%)	0.917
	With family	30 (13.0%)	201 (87.0%)	
	None	3 (14.3%)	18 (85.7%)	

Rely on someone to do daily activities	Independent	25 (21.6%)	91 (78.4%)	< 0.001
	Partially dependent	8 (7.9%)	93 (92.1%)	
	Entirely dependent	1 (2.1%)	47 (97.9%)	

Monthly income	3000 to 10,000	23 (12.2%)	166 (87.8%)	0.287
	11,000 to 20,000	6 (10.9%)	49 (89.1%)	
	> 20,000	5 (23.8%)	16 (76.2%)	

^∗^Results are expressed as frequencies and percentages.

**Table 4:** Differences in the overall quality of life score between subjects with and without chronic conditions.

Parameter	Chronic condition∗		*p* value
	No	Yes	
Diabetes	21.50 (5.76)	19.65 (6.05)	0.013
Hypertension	22.79 (5.43)	18.48 (5.71)	< 0.001
Osteoporosis	21.45 (5.74)	18.55 (6.02)	< 0.001
Eye disorder	21.37 (5.91)	19.29 (5.87)	0.005
Hypercholesterolemia	21.41 (5.79)	19.00 (6.01)	0.003
Kidney disease	21.06 (5.87)	14.24 (2.83)	< 0.001
Hearing disorder	21.07 (5.87)	18.65 (6.01)	0.006
Respiratory disease	20.92 (5.81)	18.16 (6.48)	0.012
Cardiac disease	21.10 (5.88)	18.08 (5.81)	0.001
Had ever a thrombotic event	20.96 (5.86)	18.22 (6.13)	0.009
Had a memory disorder	20.69 (5.98)	19.49 (5.86)	0.290
Cancer	20.56 (5.94)	19.59 (8.68)	0.924

^∗^Results were expressed as mean (SD).

**Table 5:** Factors associated with the scores of the quality of life.

Parameter	Category	Overall QOL		Health and functioning		Social and economic		Psychological and spiritual		Family	
		Mean (SD)	*p*	Mean (SD)	*p*	Mean (SD)	*p*	Mean (SD)	*p*	Mean (SD)	*p*
Gender	Male	20.39 (6.08)	0.742	18.89 (7.08)	0.952	20.00 (6.04)	0.816	21.72 (6.86)	0.381	23.13 (6.41)	0.922
	Female	20.64 (5.92)		18.98 (6.60)		20.19 (6.19)		22.33 (6.71)		23.32 (6.03)	

Age	60 to 69	22.07 (5.48)	< 0.001	21.03 (5.95)	< 0.001	20.99 (6.01)	0.007	23.83 (5.99)	< 0.001	23.91 (5.90)	0.083
	70 to 79	19.21 (6.15)		17.21 (6.82)		19.40 (6.15)		20.33 (7.25)		22.70 (6.29)	
	80 or more	16.35 (5.19)		12.96 (5.73)		17.60 (5.86)		17.78 (6.39)		21.36 (6.79)	

Marital status	Married	21.48 (5.71)	0.004	20.21 (6.51)	< 0.001	20.62 (6.11)	0.175	22.94 (6.42)	0.030	24.06 (5.73)	0.001
	Single	19.70 (6.03)		18.77 (6.32)		19.74 (6.41)		21.32 (6.89)		19.88 (7.06)	
	Divorced	16.63 (6.30)		14.61 (6.97)		17.44 (5.57)		18.01 (7.38)		18.95 (6.82)	
	Widow	19.45 (6.03)		16.82 (6.75)		19.63 (6.09)		21.26 (7.07)		23.58 (5.99)	

Educational level	Illiterate	20.08 (5.97)	0.004	17.89 (6.72)	< 0.001	19.80 (5.97)	0.052	21.99 (6.92)	0.034	23.67 (6.61)	0.193
	Primary school	18.77 (5.36)		16.56 (6.16)		18.97 (5.64)		20.23 (6.52)		22.06 (5.88)	
	Middle school	19.28 (6.65)		17.57 (7.03)		18.67 (6.87)		20.59 (7.80)		22.83 (6.08)	
	Secondary school	21.08 (5.58)		19.93 (6.20)		20.41 (5.79)		22.53 (6.47)		23.07 (5.74)	
	University	22.68 (5.87)		21.87 (6.67)		21.88 (6.29)		24.13 (6.01)		24.11 (6.33)	

Employment status	Employed	23.43 (5.74)	0.027	23.02 (5.37)	0.002	23.22 (5.82)	0.023	24.26 (6.51)	0.214	23.63 (7.45)	0.535
	Retired	20.92 (5.99)		19.49 (6.96)		20.44 (6.08)		22.27 (6.73)		23.55 (6.00)	
	Unemployed	19.71 (5.85)		17.73 (6.50)		19.30 (6.09)		21.58 (6.82)		22.86 (6.17)	

Living condition	With caregiver	13.48 (3.21)	< 0.001	10.52 (2.66)	< 0.001	15.08 (5.18)	0.007	14.14 (4.35)	< 0.001	17.87 (5.47)	< 0.001
	With family	21.12 (5.81)		19.55 (6.64)		20.50 (6.09)		22.73 (6.50)		23.96 (5.80)	
	None	18.55 (5.98)		17.44 (6.53)		19.07 (5.81)		20.06 (7.48)		18.69 (7.18)	

Rely on someone to do daily activities	Independent	22.50 (5.42)	< 0.001	21.72 (6.02)	< 0.001	21.31 (5.95)	0.008	24.03 (5.76)	< 0.001	24.23 (5.70)	0.091
	Partially dependent	19.79 (6.10)		17.94 (6.66)		19.66 (6.23)		21.36 (7.31)		22.65 (6.55)	
	Entirely dependent	17.41 (5.34)		14.32 (5.68)		18.20 (5.80)		18.96 (6.47)		22.12 (6.22)	

Monthly income	3000 to 10,000	20.48 (5.81)	0.931	18.69 (6.60)	0.570	20.05 (6.12)	0.958	22.22 (6.58)	0.939	23.34 (6.03)	0.984
	11,000 to 20,000	20.80 (6.29)		19.61 (7.22)		20.23 (6.10)		21.87 (7.30)		23.32 (5.90)	
	> 20,000	20.51 (6.85)		19.44 (7.43)		20.39 (6.54)		21.57 (7.22)		22.21 (8.14)	

Chronic condition	No	23.03 (5.81)	0.011	22.78 (5.95)	< 0.001	22.80 (6.18)	0.008	23.86 (6.18)	0.084	22.87 (6.55)	0.822
	Yes	20.18 (5.92)		18.38 (6.72)		19.72 (6.03)		21.84 (6.82)		23.30 (6.13)	

Number of chronic conditions	None	23.03 (5.81)	< 0.001	22.78 (5.95)	< 0.001	22.80 (6.18)	< 0.001	23.86 (6.18)	< 0.001	22.87 (6.55)	0.061
	1 to 3	21.78 (5.35)		20.53 (5.97)		20.67 (5.86)		23.56 (6.00)		24.25 (5.51)	
	4 or more	18.08 (6.01)		15.55 (6.63)		18.48 (6.05)		19.57 (7.18)		22.06 (6.68)	

**Table 6:** Results of the multivariate linear regression analysis for the predictors of quality of life among the older adults population.

Parameter	Category	Overall QOL		Health and functioning		Social and economic		Psychological and spiritual		Family	
		Beta (95% CI)	*p*	Beta (95% CI)	*p*	Beta (95% CI)	*p*	Beta (95% CI)	*p*	Beta (95% CI)	*p*
Gender	Male	—		—		—		—		—	
	Female	0.08 (−1.65–1.81)	0.928	−0.11 (−1.94 to 1.71)	0.902	0.53 (−1.40–2.46)	0.589	0.06 (−1.97–2.09)	0.953	−0.11 (−2.05 to 1.83)	0.911

Age	60 to 69	—		—		—		—		—	
	70 to 79	−1.63 (−3.36 to 0.09)	0.064	−2.12 (−3.95 to −0.29)	0.023	−0.65 (−2.58 to 1.29)	0.511	−2.61 (−4.64 to −0.58)	0.012	−0.35 (−2.29 to 1.59)	0.721
	80 or more	−2.18 (−4.88 to 0.51)	0.112	−3.51 (−6.36 to −0.66)	0.016	−0.25 (−3.27 to 2.77)	0.872	−3.11 (−6.28 to 0.06)	0.054	−0.34 (−3.37 to 2.69)	0.824

Marital status	Married	—		—		—		—		—	
	Single	−2.00 (−4.69 to 0.69)	0.145	−2.13 (−4.98 to 0.72)	0.142	−1.25 (−4.26 to 1.77)	0.416	−1.49 (−4.65 to 1.68)	0.356	−3.50 (−6.53 to −0.48)	0.023
	Divorced	−2.03 (−4.96 to 0.89)	0.172	−2.81 (−5.90 to 0.28)	0.074	−0.75 (−4.03 to 2.52)	0.650	−1.80 (−5.24 to 1.64)	0.304	−2.11 (−5.39 to 1.18)	0.207
	Widow	0.88 (−1.22–2.98)	0.408	0.08 (−2.14–2.30)	0.943	1.37 (−0.98–3.72)	0.253	1.44 (−1.02–3.91)	0.250	1.51 (−0.84–3.87)	0.207

Educational level	Illiterate	—		—		—		—		—	
	Primary school	−1.14 (−3.37 to 1.08)	0.313	−1.56 (−3.92 to 0.79)	0.193	−0.47 (−2.96 to 2.03)	0.712	−1.29 (−3.91 to 1.33)	0.333	−1.05 (−3.55 to 1.46)	0.410
	Middle school	−1.51 (−4.05 to 1.03)	0.242	−1.57 (−4.26 to 1.11)	0.250	−1.53 (−4.38 to 1.31)	0.289	−1.98 (−4.96 to 1.01)	0.193	−0.71 (−3.56 to 2.14)	0.625
	Secondary school	−2.02 (−4.46 to 0.42)	0.105	−2.38 (−4.96 to 0.20)	0.070	−1.32 (−4.06 to 1.41)	0.341	−2.21 (−5.08 to 0.66)	0.130	−1.87 (−4.61 to 0.88)	0.181
	University	−0.38 (−3.09 to 2.33)	0.781	−1.11 (−3.97 to 1.76)	0.448	0.45 (−2.59–3.48)	0.773	−0.20 (−3.39 to 2.99)	0.902	0.08 (−2.96–3.13)	0.958

Employment status	Employed	—		—		—		—		—	
	Retired	−1.72 (−4.46 to 1.02)	0.217	−2.08 (−4.97 to 0.81)	0.158	−2.61 (−5.68 to 0.46)	0.095	−1.16 (−4.37 to 2.06)	0.480	−0.10 (−3.17 to 2.98)	0.950
	Unemployed	−3.05 (−6.10 to −0.01)	0.049	−3.32 (−6.54 to −0.10)	0.043	−4.33 (−7.74 to −0.92)	0.013	−2.31 (−5.89 to 1.27)	0.206	−1.36 (−4.78 to 2.06)	0.435

Living condition	With caregiver	—		—		—		—		—	
	With family	3.90 (0.15–7.65)	0.041	2.56 (−1.40–6.52)	0.205	4.02 (−0.18–8.22)	0.060	5.22 (0.81–9.62)	0.021	5.49 (1.27–9.70)	0.011
	None	2.44 (−1.97–6.86)	0.277	1.86 (−2.81–6.52)	0.434	2.93 (−2.02–7.88)	0.244	3.93 (−1.27–9.12)	0.138	1.33 (−3.63–6.29)	0.597

Rely on someone to do daily activities	Independent	—		—		—		—		—	
	Partially dependent	−1.63 (−3.16 to −0.10)	0.036	−2.24 (−3.86 to −0.62)	0.007	−0.81 (−2.52 to 0.91)	0.354	−1.58 (−3.38 to 0.22)	0.086	−1.35 (−3.07 to 0.37)	0.124
	Entirely dependent	−1.62 (−3.88 to 0.64)	0.160	−2.76 (−5.15 to −0.37)	0.024	−0.29 (−2.83 to 2.24)	0.819	−1.64 (−4.30 to 1.02)	0.225	−0.62 (−3.16 to 1.92)	0.630

Monthly income	3000 to 10,000	—		—		—		—		—	
	11,000 to 20,000	−0.57 (−2.38 to 1.24)	0.534	−0.09 (−2.00 to 1.82)	0.927	−0.67 (−2.70 to 1.35)	0.514	−1.39 (−3.51 to 0.74)	0.200	−0.48 (−2.51 to 1.55)	0.639
	> 20,000	−0.81 (−3.40 to 1.79)	0.541	−0.12 (−2.86 to 2.63)	0.932	−0.80 (−3.71 to 2.11)	0.589	−1.55 (−4.60 to 1.50)	0.318	−1.32 (−4.24 to 1.60)	0.374

Chronic conditions	Diabetes	0.57 (−0.94–2.07)	0.458	0.15 (−1.44–1.74)	0.857	1.12 (−0.56–2.81)	0.190	0.72 (−1.05–2.49)	0.424	0.63 (−1.06–2.32)	0.465
	Hypertension	−2.08 (−3.65 to −0.50)	0.010	−2.73 (−4.40 to −1.06)	0.001	−1.62 (−3.39 to 0.15)	0.072	−1.86 (−3.71 to 0.00)	0.050	−1.39 (−3.16 to 0.38)	0.124
	Osteoporosis	−1.08 (−2.69 to 0.53)	0.188	−0.85 (−2.56 to 0.86)	0.328	−1.41 (−3.22 to 0.40)	0.125	−1.52 (−3.42 to 0.38)	0.116	−0.55 (−2.37 to 1.26)	0.547
	Eye disorder	−0.76 (−2.23 to 0.70)	0.307	−1.04 (−2.59 to 0.51)	0.188	−0.71 (−2.35 to 0.93)	0.396	−0.43 (−2.16 to 1.29)	0.620	−0.52 (−2.16 to 1.13)	0.536
	HCLM	−1.16 (−2.72 to 0.39)	0.141	−1.64 (−3.28 to 0.00)	0.051	−0.54 (−2.28 to 1.20)	0.544	−1.11 (−2.94 to 0.72)	0.233	−0.83 (−2.58 to 0.92)	0.350
	Kidney disease	−3.93 (−6.58 to −1.28)	0.004	−3.74 (−6.54 to −0.93)	0.009	−4.52 (−7.50 to −1.55)	0.003	−3.96 (−7.08 to −0.84)	0.013	−3.47 (−6.45 to −0.49)	0.023
	Hearing disorder	−0.15 (−1.95 to 1.65)	0.867	−0.43 (−2.33 to 1.48)	0.659	0.42 (−1.60–2.43)	0.684	−0.28 (−2.39 to 1.84)	0.797	−0.12 (−2.14 to 1.91)	0.911
	Respiratory disease	−1.02 (−2.98 to 0.94)	0.305	−1.81 (−3.88 to 0.27)	0.087	−1.06 (−3.26 to 1.13)	0.341	−0.19 (−2.50 to 2.11)	0.869	−0.01 (−2.21 to 2.19)	0.993
	Cardiac disease	−0.94 (−2.95 to 1.07)	0.357	−0.31 (−2.43 to 1.82)	0.777	−1.85 (−4.11 to 0.40)	0.107	−1.21 (−3.58 to 1.15)	0.314	−0.89 (−3.15 to 1.37)	0.440
	A thrombotic event	1.23 (−1.07–3.53)	0.294	0.01 (−2.42–2.44)	0.995	1.90 (−0.68–4.48)	0.148	2.37 (−0.34–5.07)	0.086	1.82 (−0.76–4.40)	0.167
	A memory disorder	−0.31 (−2.61 to 2.00)	0.794	−0.35 (−2.79 to 2.09)	0.779	−1.23 (−3.81 to 1.36)	0.351	0.89 (−1.83–3.60)	0.520	−0.31 (−2.90 to 2.28)	0.812
	Cancer	−2.94 (−8.45 to 2.56)	0.293	−3.73 (−9.55 to 2.08)	0.207	−2.28 (−8.45 to 3.88)	0.467	−2.48 (−8.95 to 3.99)	0.451	−2.58 (−8.77 to 3.60)	0.411

Abbreviation: HCLM, hypercholesterolemia.

## Data Availability

All data are available upon contact with the corresponding author.
